# Serial Embolic Events From Left Atrial Appendage Thrombus Detected After Mechanical Thrombectomy: A Case Report and Management Considerations

**DOI:** 10.7759/cureus.95202

**Published:** 2025-10-22

**Authors:** Satoshi Horiguchi, Yoshinori Maki, Yusuke Kobayashi, Yuto Mitsuno, Ryotaro Otsuka

**Affiliations:** 1 Department of Neurosurgery, Nagahama City Hospital, Nagahama, JPN; 2 Department of Neurosurgery, Hikone Chuo Hospital, Hikone, JPN; 3 Department of Cardiology, Nagahama City Hospital, Nagahama, JPN; 4 Department of Neurosurgery, Kyoto University Graduate School of Medicine, Kyoto, JPN

**Keywords:** atrial fibrillation (af), cerebral thrombosis, endovascular mechanical thrombectomy, left atrial appendage occlusion, left atrial appendage thrombus, recurrent arterial thrombosis

## Abstract

Atrial fibrillation (AF) is a major cause of ischemic stroke, with the left atrial appendage (LAA) serving as the predominant source of thromboembolism. While the association between AF, LAA thrombus, and cerebral embolism is well established, direct visualization of an LAA thrombus in the acute post-thrombectomy setting, followed by recurrent large vessel occlusion when anticoagulation is withheld, has been only rarely documented.

We report the case of an 82-year-old woman with AF on edoxaban who presented with acute right internal carotid artery (ICA) occlusion. Mechanical thrombectomy achieved successful reperfusion (TICI 2b). Subsequent imaging revealed right basal ganglia infarction with minor hemorrhagic transformation, accompanied by partial neurological recovery. Two days after onset, contrast-enhanced chest CT unexpectedly demonstrated an LAA thrombus, prompting initiation of intravenous heparin. On day 5, the patient developed sudden impaired consciousness, and MRI revealed left ICA occlusion. Repeat thrombectomy achieved partial reperfusion (TICI 2a), but follow-up imaging showed extensive left hemispheric infarction. Despite intensive treatment, she was left with severe disability (modified Rankin Scale score 5) and was transferred to a rehabilitation facility after 68 days.

This case highlights the clinical importance of detecting LAA thrombus on contrast-enhanced CT after thrombectomy. Anticoagulation could not be promptly resumed due to hemorrhagic transformation, and the patient subsequently experienced recurrent embolic events. These findings underscore the need for thorough evaluation of potential embolic sources after thrombectomy, thoughtful consideration of when and how to restart anticoagulation, and the possible role of LAA occlusion as a secondary prevention strategy.

## Introduction

Atrial fibrillation (AF) is one of the most common cardiac arrhythmias and is strongly associated with cardioembolic ischemic stroke. The left atrial appendage (LAA) is the predominant site of thrombus formation in AF patients, accounting for up to 90% of embolic sources in non-valvular AF [1]. Anticoagulation with either vitamin K antagonists or direct oral anticoagulants substantially reduces, but does not eliminate, the risk of embolic events [2].

Mechanical thrombectomy (MT) has revolutionized the treatment of large-vessel occlusion (LVO), significantly improving functional outcomes [3]. However, patients with AF remain at increased risk of recurrent embolic events, particularly when active thrombus persists in the LAA. A recent study revealed that LAA thrombus was identified by CT angiography in approximately 4% of patients with stroke or transient ischemic attack, and the presence of LAA thrombus was associated with poorer three-month outcomes on the modified Rankin Scale (mRS) [4]. Such direct visualization of LAA thrombus after MT is extremely rare and clinically significant for therapeutic decision-making.

When LAA thrombus is identified, anticoagulation therapy is the first-line strategy, as thrombus resolution is achieved in most cases. However, when hemorrhagic transformation occurs after MT, resuming anticoagulation becomes a major clinical dilemma, requiring careful timing and individualized management.

Here, we report a case of recurrent LVO occurring within days of initial thrombectomy. The patient’s LAA thrombus was visualized on contrast-enhanced CT after the first stroke. Anticoagulation could not be reinitiated promptly due to hemorrhagic transformation, and the patient subsequently developed contralateral internal carotid artery (ICA) occlusion. This case underscores the clinical challenges of managing patients with visible LAA thrombus after thrombectomy and highlights the need for effective strategies to prevent recurrent embolic stroke.

## Case presentation

**Figure 1:**
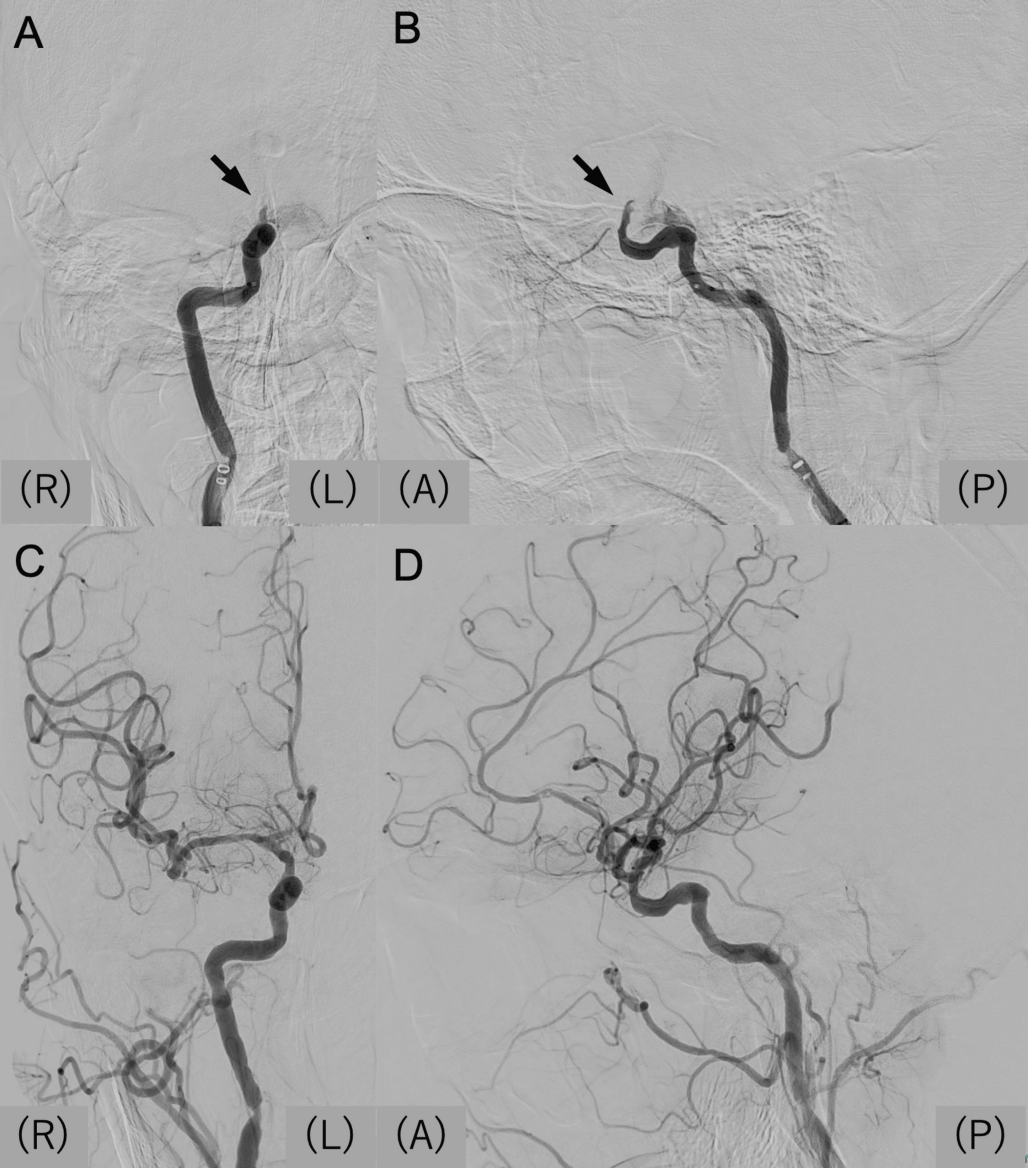
First mechanical thrombectomy (A, B) Preoperative angiography obtained from a microcatheter positioned proximal to the occlusion site. Anteroposterior (A) and lateral (B) views demonstrate right ICA occlusion at the C1 segment (arrows). (C, D) Postoperative angiography shows successful recanalization of the right ICA. ICA: internal carotid artery; (A): anterior; (P): posterior; (R): right; (L): left

An 82-year-old woman with a history of hypertension and AF, treated with 30 mg of edoxaban daily, was found collapsed at home by her family. On arrival, she presented with left hemiparesis (MRC grade 1/5), mild dysarthria, and left visual inattention, corresponding to an NIHSS score of 22. Her CHA₂DS₂-VASc Score was 6, and HAS-BLED score was 3. The time from the last known well to hospital admission was five hours. Electrocardiography confirmed AF. Magnetic resonance imaging (MRI) demonstrated an acute infarction due to right ICA occlusion. MT was performed using a stent retriever (EMBOTRAP III, Johnson & Johnson MedTech, New Brunswick, NJ, USA) and an aspiration catheter (EMBOVAC, Johnson & Johnson MedTech), achieving successful reperfusion with a Thrombolysis in Cerebral Infarction (TICI) score of 2b (Figure 1). Puncture-to-reperfusion time was 26 minutes.

**Figure 2:**
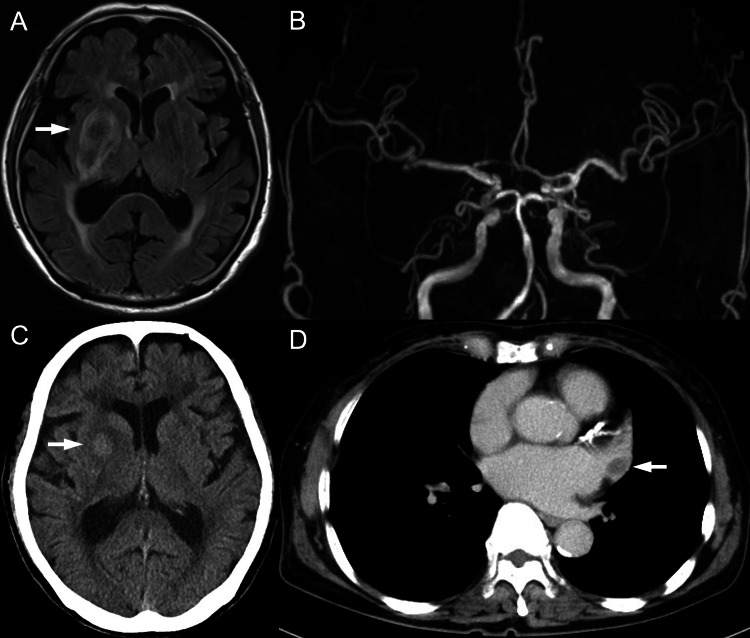
Post-procedural CT and MR images (A) MRI FLAIR sequence demonstrating an ischemic lesion with hemorrhagic transformation in the right basal ganglia (arrow). (B) MRA confirming persistent recanalization of the right ICA. (C) Head CT showing hemorrhagic changes in the right basal ganglia (arrow). (D) Contrast-enhanced chest CT revealing a filling defect in the LAA, consistent with thrombus (arrow). MRI: magnetic resonance imaging; FLAIR: fluid-attenuated inversion recovery; MRA: magnetic resonance angiography; ICA: internal carotid artery; LAA: left atrial appendage

On the following day, CT and MRI revealed right basal ganglia infarction with minor hemorrhagic transformation (Figure 2A, 2C). Magnetic resonance angiography (MRA) confirmed persistent recanalization (Figure 2B). Her neurological deficits partially improved, with left-sided weakness recovering to grade 3/5. Because resuming anticoagulation in the setting of minor hemorrhagic transformation was considered to carry a high risk of bleeding, treatment was withheld according to the AHA/ASA guideline [5].

On day 2 after onset, contrast-enhanced chest CT was performed to rule out aortic dissection, which had been suspected on preoperative MRA. Unexpectedly, CT revealed a thrombus in the LAA (Figure 2D). In consultation with cardiology, although a vitamin K antagonist was recommended for anticoagulation, continuous intravenous heparin infusion was chosen instead to minimize the bleeding risk associated with hemorrhagic transformation. Heparin was initiated at a dose of 10,000 IU per 24 hours, with a target activated partial thromboplastin time (APTT) range of 45-60 seconds. APTT was monitored daily to guide dose adjustment, rising from 29.8 seconds on admission to 35.3 seconds by day 4.

**Figure 3:**
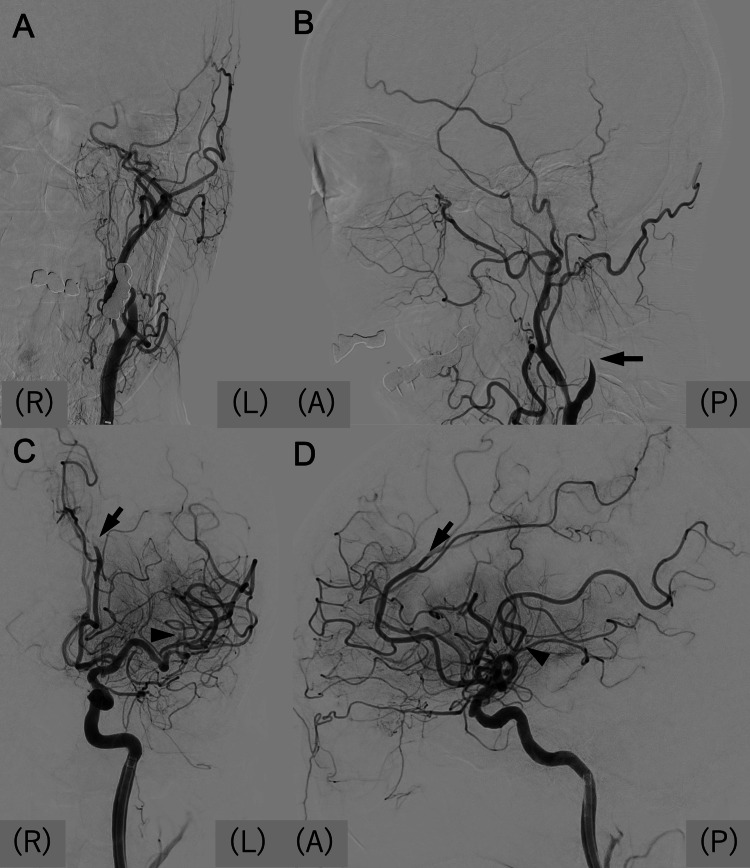
Mechanical thrombectomy for recurrent embolic event (A, B) Preoperative left carotid angiography in anteroposterior (A) and lateral (B) views showing left ICA occlusion (arrow in B). (C, D) Postoperative angiography demonstrating partial recanalization of the left ICA, with persistent occlusion of the left M2 (arrowheads) and A3 (arrows) segments. ICA: internal carotid artery; (A): anterior; (P): posterior; (R): right; (L): left

On day 5, the patient experienced sudden impairment of consciousness. Recurrence of embolism or enlargement of the right basal ganglia hemorrhage under heparinization was suspected, and emergent imaging was obtained. MRA revealed acute left ICA occlusion. Urgent thrombectomy was performed using a stent retriever (EMBOTRAP III, Johnson & Johnson MedTech) and an aspiration catheter (EMBOVAC, Johnson & Johnson MedTech), resulting in partial reperfusion (TICI 2a) (Figure 3). Puncture-to-reperfusion time was 110 minutes.

**Figure 4:**
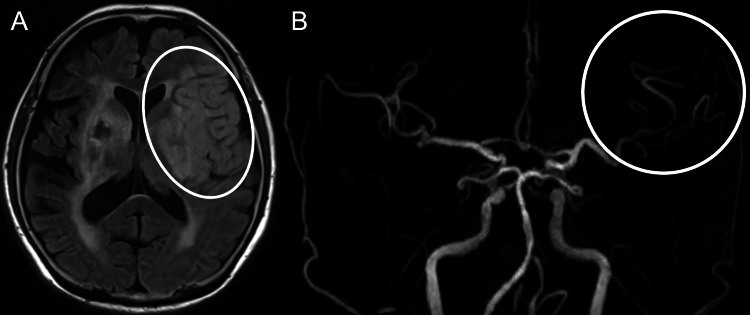
MR images after second mechanical thrombectomy (A) MRI FLAIR sequence showing an ischemic lesion in the left frontal lobe (oval). (B) MRA revealing partial recanalization of the left MCA (circle). MRI: magnetic resonance imaging; FLAIR: fluid-attenuated inversion recovery; MRA: magnetic resonance angiography; MCA: middle cerebral artery

Follow-up MRI demonstrated extensive left frontal and parietal infarction (Figure 4). Despite maximal supportive care, her condition did not improve, leaving her with profound neurological deficits. At discharge, her Glasgow Coma Scale score was 4 (E1V1M2), corresponding to a modified Rankin Scale (mRS) score of 5. After 68 days of hospitalization, she was discharged to a long-term rehabilitation facility.

The patient timeline is given in Table 1.

**Table 1:** Patient Timeline ICA: internal carotid artery; APTT: activated partial thromboplastin time; TICI: thrombolysis in cerebral infarction; MRA: magnetic resonance angiography; mRS: modified Rankin Scale; GCS: Glasgow Coma Scale.

Day	Events
Day 0 (Onset)	An 82-year-old woman with hypertension and atrial fibrillation (AF) on edoxaban 30 mg presented with left hemiparesis (MRC 1/5), mild dysarthria, and left visual inattention (NIHSS 22). MRI revealed acute infarction due to right ICA occlusion. APTT was 29.8 seconds. Mechanical thrombectomy (MT) was performed with successful reperfusion (TICI 2b). Puncture-to-reperfusion time: 26 minutes.
Day 1	CT and MRI showed right basal ganglia infarction with minor hemorrhagic transformation. MRA confirmed persistent recanalization. Neurological deficits partially improved (left weakness grade 3/5). Anticoagulation was withheld due to hemorrhagic risk.
Day 2	Contrast-enhanced chest CT revealed thrombus in the left atrial appendage (LAA). Continuous intravenous heparin infusion was started after cardiology consultation.
Day 4	Heparin therapy continued; APTT increased to 35.3 seconds. No recurrent neurological events were observed.
Day 5	Sudden loss of consciousness occurred. MRA revealed acute left ICA occlusion. Urgent thrombectomy was performed, achieving partial reperfusion (TICI 2a). Puncture-to-reperfusion time: 110 minutes.
Day 6	MRI showed extensive left frontal and parietal infarction.
Day 68	Discharged to a long-term rehabilitation facility, mRS 5, GCS 4 (E1V1M2).

## Discussion

In this case, recurrent cardioembolic strokes due to AF were complicated by the presence of an LAA thrombus and cerebral hemorrhagic transformation after initial MT. The clinical course highlighted the central challenge of balancing the need for early, aggressive anticoagulation against the heightened risk of intracranial bleeding. This scenario also raised the question of whether direct intervention for the LAA thrombus should be considered during the acute phase of cerebral infarction.

AF-related embolism is well recognized as a leading mechanism of LVO. In this context, the LAA serves as the principal site of thrombus formation, which directly relates to the risk of recurrent embolic events, as demonstrated in our patient [1]. Therefore, detection of thrombus in the LAA is clinically significant.

The American Heart Association/American Stroke Association guidelines recommend transthoracic echocardiography (TTE) for routine cardiac evaluation after stroke, including in patients with AF, primarily to assess cardiac function and screen for structural sources of embolism [5]. Transesophageal echocardiography (TEE) or cardiac CT may be considered if TTE is nondiagnostic or if there is clinical suspicion of additional cardiac sources of embolism that would change management, such as LAA thrombus, valvular pathology, or aortic arch disease [5-7]. However, in patients with known AF, the index stroke itself is an indication for anticoagulation, and routine use of TEE or cardiac CT generally does not alter management or improve outcomes, as supported by observational data [8].

TEE has higher sensitivity for detecting LAA thrombus compared with TTE, but its use should be individualized based on the clinical context, such as unexplained recurrent embolism, suspicion of additional cardiac pathology, or when findings would influence therapeutic decisions [7]. Cardiac CT is emerging as a sensitive alternative for thrombus detection but is not yet standard for all patients with AF and stroke [9,10]. In our patient, contrast-enhanced chest CT was planned to rule out aortic dissection, and TEE was not performed in the acute stage of stroke due to its procedural burden. TTE was planned as part of routine evaluation.

Although not standard practice, pre-procedural cardiac CT is sometimes performed in acute ischemic stroke or AF cohorts, where detection of intracardiac thrombus, including LAA thrombus, is not uncommon. In hyperacute multimodal computed tomography angiography (CTA) studies, the prevalence of LAA thrombus has been reported as 14-15% among patients with known AF and was significantly associated with intracranial LVO [9,11]. A recent study further revealed that LAA thrombus was identified by CTA in 10.5% of AF patients with stroke or transient ischemic attack, and its presence was associated with poorer three-month outcomes [4].

Beyond embolic source detection, large multicenter analyses have demonstrated that ischemic core volume strongly modifies the effect of reperfusion therapy, underscoring the clinical value of baseline perfusion imaging such as CT perfusion (CTP) in guiding acute stroke management [12]. Therefore, pre-procedural contrast-enhanced CT and CTP could be considered complementary tools for both embolic source assessment and tissue viability evaluation. However, these potential benefits must be balanced against the inherent time constraints in the hyperacute stroke setting. In our case, MT was prioritized over extended CTA and CTP to expedite reperfusion and minimize delay in recanalization therapy.

When LAA thrombus is identified, intensification or adjustment of oral anticoagulation is typically attempted as a first-line strategy, as thrombus resolution is achieved in most cases. However, this approach carries a significant bleeding risk and may not be effective in all patients [13]. Bleeding complications are particularly detrimental, as they not only promote a hypercoagulable state but also delay the initiation of timely secondary prophylaxis against thrombosis. This dual impact underscores the importance of aggressive bleed-avoidance strategies, especially in patients with existing thrombotic burdens such as LAA thrombus, intra-atrial thrombi, or deep vein thrombosis (DVT).

In patients with contraindications to intensified or continued anticoagulation - or with persistent LAA thrombus despite adequate anticoagulation - LAA occlusion (LAAO) may be considered. Early case reports described the use of cerebral embolic protection during LAAO in patients with persistent thrombus [14]. Importantly, the presence of intracardiac thrombus, particularly within the atrium, is generally regarded as a contraindication for LAAO device implantation. Nonetheless, recent case series have demonstrated the feasibility and safety of percutaneous mechanical aspiration of thrombus, sometimes followed by immediate LAAO, with cerebral protection devices used to minimize periprocedural embolic risk [15,16]. In such high-risk patients, LAAO may offer a mechanical solution to prevent recurrent embolic events when anticoagulation alone is insufficient or contraindicated. In our patient, however, the application of LAA thrombectomy followed by LAAO remains debatable due to intracerebral hemorrhagic complications.

Another important challenge with this approach is determining the optimal timing of intervention. A prospective study reported that the cumulative risk of recurrent ischemic events - including stroke, transient ischemic attack, and systemic embolism - was 2.7% at 14 days and 5.1% at 30 days, with most events occurring early in the post-stroke period [17]. This early recurrence risk is notably higher than the average annualized recurrence rates reported in longer-term studies and meta-analyses, which typically range from 3.75% to 4.3% per year in patients with atrial fibrillation and prior stroke, even with widespread use of oral anticoagulants [18,19]. The risk is greatest in the first two weeks after stroke, underscoring the importance of timely initiation of secondary prevention strategies. Recent randomized controlled trials, including the TIMING [20] and ELAN [21] studies, demonstrated that early initiation of direct oral anticoagulants after ischemic stroke was as safe as, and potentially more effective than, delayed initiation, supporting consideration of early anticoagulation in eligible patients. In our case, recurrence occurred on day 5. After the first MT, escalation of anticoagulation therapy was avoided due to hemorrhagic transformation. When anticoagulation is unsafe or ineffective, contemporary evidence suggests that MT for cerebral arteries along with immediate LAAO, with or without LAA thrombectomy, may be considered in selected cases, potentially using specialized techniques to mitigate recurrent embolic risk. In retrospect, the development of hemorrhagic transformation was a pivotal factor that limited therapeutic options. Without hemorrhage, intensification of anticoagulation might have been feasible and could have reduced the risk of recurrent infarction, though this remains speculative.

Clinical implications in this case

In this case, the development of hemorrhagic transformation after the first thrombectomy precluded early escalation of anticoagulation, despite the presence of a visible LAA thrombus. This limitation illustrates a common clinical dilemma in patients with atrial fibrillation who experience both thrombotic and hemorrhagic events. When anticoagulation cannot be safely intensified, LAAO may theoretically serve as a mechanical alternative to prevent further embolic events. However, performing LAAO in the presence of intracardiac thrombus or recent intracerebral hemorrhage carries substantial procedural and bleeding risks. Therefore, the decision to pursue LAAO should be made cautiously, guided by individual patient factors, multidisciplinary discussion, and evolving evidence. In our case, the presence of intracranial bleeding rendered LAAO unsuitable in the acute phase, highlighting the need for safer strategies for stroke prevention in this complex clinical scenario.

## Conclusions

We reported a case of recurrent contralateral ICA occlusion shortly after successful thrombectomy, in which an incidental LAA thrombus was identified. Anticoagulation could not be reinitiated promptly due to hemorrhagic transformation, and the patient subsequently developed devastating recurrent embolic events. This case underscores the need for individualized decision-making on the timing of anticoagulation resumption and highlights that early multimodality cardiac imaging - such as cardiac CT - after thrombectomy may help identify LAA thrombus and guide tailored management. While LAA occlusion may be considered in selected cases, its application should be guided by patient-specific factors and current evidence.
